# Correction: Protein patterns template arrays of magnetic nanoparticles

**DOI:** 10.1039/c9ra90085c

**Published:** 2019-11-20

**Authors:** Srikanth Nayak, Honghu Zhang, Xunpei Liu, Shuren Feng, Pierre Palo, Marit Nilsen-Hamilton, Mufit Akinc, Surya Mallapragada

**Affiliations:** Division of Materials Science and Engineering, Ames Laboratory Ames IA 50011 USA suryakm@iastate.edu; Department of Chemical & Biological Engineering, Iowa State University Ames Iowa 50011 USA; Department of Materials Science & Engineering, Iowa State University Ames Iowa 50011 USA; Roy J. Carver Department of Biochemistry, Biophysics and Molecular Biology, Iowa State University Ames Iowa 50011 USA

## Abstract

Correction for ‘Protein patterns template arrays of magnetic nanoparticles’ by Srikanth Nayak *et al.*, *RSC Adv.*, 2016, **6**, 57048–57056.

The authors regret that two sentences in the introduction of the original article did not fully represent the previous work cited in the article.

On page 57048, the sentence, “Mms6, an amphiphilic protein found in the magnetosomes of *Magnetospirillum magneticum*, can control the size, shape and monodispersity of magnetite nanoparticles, both *in vivo*^9,10^ and *in vitro*^11,12^ using a room temperature co-precipitation (RTCP) method.” should be changed to “Mms6, an amphiphilic protein found in the magnetosomes of *Magnetospirillum magneticum*, can control the size, shape and monodispersity of magnetite nanoparticles, both *in vivo* and *in vitro* using a room temperature co-precipitation (RTCP) method”.^9–12^ This corrected sentence is a better representation of the prior work cited here. In addition, on page 57049, the sentence, “No control groups for studying the role of immobilized proteins on the formation of magnetite nanoparticles were considered in these studies.” should be changed to “Very few studies investigating the role of immobilized proteins on the formation of magnetite nanoparticles were conducted, that included appropriate immobilized protein controls”.

In the supplementary information of ref. 30, a single image shows surface mineralization of magnetic nanoparticles (MNPs) on an immobilized bovine serum albumin (BSA) control, but to a lesser extent than on Mms6. No detailed analysis of these particles was provided. BSA is also structurally different from Mms6 and not as good a control as the mutant m2Mms6 used in our study, which forms micelles like Mms6 (ref. 18) but does not bind iron to high capacity (ref. 11).

The Royal Society of Chemistry apologises for these errors and any consequent inconvenience to authors and readers.

## Supplementary Material

